# Stress Echocardiography in the Diagnosis and Evaluation of Pulmonary Hypertension: Practical Recommendations, Haemodynamic Phenotyping, and Application in Adults and Children

**DOI:** 10.3390/diagnostics16050792

**Published:** 2026-03-06

**Authors:** Dafni Charisopoulou, George Koulaouzidis, Panagiota Kleitsioti, Nikolaos Antoniou, Christos Mantzios, Orestis Grammenos, Sotiria Iliopoulou

**Affiliations:** 1 2nd Department of Paediatrics, AHEPA University General Hospital, School of Medicine, Aristotle University of Thessaloniki, 54636 Thessaloniki, Greece; 2 Department of Biochemical Sciences, Pomeranian Medical University, 70-204 Szczecin, Poland; koulaou@yahoo.co.uk; 3 Cardiology Department, General Hospital G. Papanikolaou, 57010 Thessaloniki, Greece; pennykleitsioti@yahoo.com (P.K.); nikosn771@gmail.com (N.A.); orestes1313@hotmail.com (O.G.); sotiria.ili26@gmail.com (S.I.); 4 First Department of Cardiology, AHEPA University Hospital of Thessaloniki, 54636 Thessaloniki, Greece; chrismantzios1994@gmail.com

**Keywords:** stress echocardiography, pulmonary hypertension, exercise haemodynamics, pressure–flow relationship, unexplained dyspnea, children

## Abstract

Pulmonary hypertension (PH) is a complex condition in which early diagnosis remains challenging, particularly in patients with exertional symptoms and normal or borderline resting haemodynamics. Although right heart catheterisation is the diagnostic gold standard, transthoracic echocardiography is the recommended first-line non-invasive test. However, resting echocardiography provides only a static assessment and may underestimate disease severity in early or latent pulmonary vascular disease due to preserved pulmonary vascular compliance and adaptive right ventricular responses. Because pulmonary haemodynamics are intrinsically flow-dependent, pathological abnormalities may only emerge during increased cardiac output. Stress echocardiography, performed using exercise or pharmacological stress, enables dynamic evaluation of pulmonary pressure responses, cardiac output augmentation, right ventricular contractile reserve, and ventricular interaction. Increasing evidence indicates that stress echocardiography can unmask abnormal pulmonary pressure–flow relationships, impaired pulmonary vascular reserve, and reduced right ventricular–pulmonary arterial coupling that are not apparent at rest, thereby improving functional and haemodynamic characterisation in selected patients. This Diagnostic Review outlines the physiological basis for stress echocardiographic assessment of pulmonary circulation, proposes practical recommendations for patient selection and testing protocols, and provides a framework for interpretation centered on pressure–flow relationships rather than absolute pulmonary pressure thresholds. Particular attention is given to clinical scenarios with high diagnostic yield, including unexplained exertional dyspnoea, systemic sclerosis, suspected heart failure with preserved ejection fraction, at-risk relatives of patients with pulmonary arterial hypertension, selected athletes, and paediatric populations. Stress echocardiography should not be considered a standalone diagnostic test for PH but, when performed in experienced centers and integrated within structured diagnostic pathways, it represents a valuable non-invasive adjunct to guide referral for invasive haemodynamic confirmation.

## 1. Introduction

Pulmonary hypertension (PH) is a complex and progressive disorder characterized by elevated pulmonary arterial pressure and increased pulmonary vascular resistance, leading to right heart failure and significant morbidity and mortality [1]. PH is defined by a mean pulmonary arterial pressure (mPAP) > 20 mmHg at rest, with further classification based on pulmonary vascular resistance (PVR) and pulmonary arterial wedge pressure (PAWP) [1,2]. Despite advances in therapy, delayed diagnosis remains common and is associated with worse clinical outcomes [1].

Traditionally, right heart catheterisation (RHC) remains the gold standard method for diagnosing PH and differentiating its subtypes [1]. However, its invasive nature, limited availability, and procedure-related risks restrict its use as a widespread diagnostic tool. Transthoracic echocardiography (TTE) is therefore recommended as the initial non-invasive test in suspected PH, enabling estimation of PH probability, assessment of right ventricular (RV) structure and function, and identification of underlying cardiac causes [1,3].

However, resting TTE provides only a static assessment of cardiopulmonary haemodynamics. In the early or latent stages of pulmonary vascular disease, resting parameters (e.g., estimated pulmonary pressures, RV systolic function) may remain normal or borderline due to preserved vascular reserve and adaptive RV responses (1–4). As a result, patients with significant exertional symptoms and functional limitation may present with inconclusive or even normal resting echocardiographic findings, contributing to delayed invasive evaluation [1,4].

Pulmonary haemodynamics are flow-dependent; therefore, early abnormalities may remain undetected at rest and only manifest under conditions of increased cardiac output, such as during exercise [1,4,5]. Exercise-induced pulmonary pressure elevations can signal early pulmonary vascular disease, left heart disease, or mixed mechanisms. In this context, stress echocardiography—performed using physiological exercise or pharmacological stress—provides a non-invasive, dynamic evaluation of pulmonary pressure responses, cardiac output augmentation, and RV functional reserve [6,7].

Over the past decade, growing evidence has demonstrated that stress echocardiography can identify abnormal pressure–flow relationships, unmask impaired right ventricular–pulmonary arterial coupling, and provide incremental diagnostic information beyond resting echocardiography [5,6,7]. Importantly, contemporary guidelines have moved away from fixed exercise pressure cut-offs and toward emphasis on pressure relative to flow, cardiac output adequacy, and RV reserve [1].

This review provides practical recommendations for the diagnostic use of stress echocardiography in PH, focusing on patient selection, protocols, interpretation, integration into diagnostic pathways, and considerations for adults and children.

## 2. Baseline Echocardiographic Assessment Prior to Stress Echocardiography

Resting TTE is the first-line non-invasive test in suspected PH. It is essential for estimating the echocardiographic probability of PH, evaluating right ventricular size and function, and identifying possible left heart or valvular causes [8,9].

**Figure 1:**
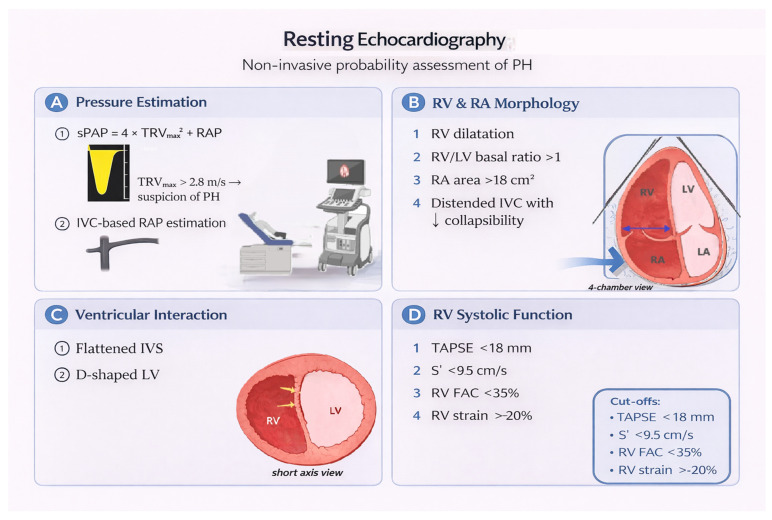
Resting Echocardiography. Non-invasive assessment in suspected pulmonary hypertension: (**A**) Estimation of sPAP based on peak TRV, using the simplified Bernoulli equation (sPAP = 4 × TRV_max_^2^ + RAP). A peak TRV > 2.8 m/s raises suspicion of PH, although pressure estimation may be affected by technical and physiological factors. (**B**) Right heart structural remodeling, including RV dilation and hypertrophy, resulting in an increased RV-to- left ventricular (LV) basal diameter ratio (>1.0) in the apical 4-chamber view, and RA enlargement. Additional echocardiographic signs of elevated right-sided pressures, including IVC dilatation with reduced inspiratory collapse, reflecting increased RAP. (**C**) Ventricular interdependence manifested by interventricular septal flattening due to chronic RV pressure overload, producing the characteristic “D-shaped” LV in the parasternal short-axis view. (**D**) Assessment of RV systolic function at rest, including TAPSE (<18 mm), tissue Doppler–derived S′ (<9.5 cm/s), RV FAC (<35%), and RV strain (>20%).

A comprehensive resting study before stress testing should include (Figure 1): tricuspid regurgitation velocity (TRV), RV dimensions and systolic function, right atrial (RA) size, inferior vena cava (IVC) parameters (for RA pressure estimation), interventricular septal shape, left ventricular systolic/diastolic function, and valvular assessment [8,9].

## 3. Physiological Determinants of Pulmonary Pressure and Implications for Interpretation

Pulmonary artery pressure is intrinsically flow-dependent and influenced by multiple physiological and constitutional factors [1,8]. Conditions associated with increased cardiac output, such as anemia, hyperthyroidism, or systemic arteriovenous shunts, may lead to elevated pulmonary pressures in the absence of intrinsic pulmonary vascular disease [10,11]. Accordingly, mildly increased TRV (>2.8 m/s) may occur in otherwise healthy individuals at rest and should not be interpreted in isolation as diagnostic of pulmonary hypertension.

Moreover, a proportion of healthy individuals exhibit an exaggerated pulmonary pressure response to exercise or hypoxia despite normal pulmonary vascular structure and function. In athletes, pulmonary artery pressure commonly rises during high workloads as a consequence of marked increases in cardiac output and, in some cases, elevated left ventricular filling pressures [12,13]. In these individuals, a compliant pulmonary vascular bed accommodates increased flow through capillary recruitment and distension, thereby maintaining low pulmonary vascular resistance.

Finally, age and body mass index (BMI) are key determinants of pulmonary arterial pressure (PAP) [14,15]. Pulmonary pressures tend to rise with age, largely due to the loss of pulmonary vascular compliance and increased stiffness. In individuals over 50 years old, the upper limit of normal for systolic PAP is higher than in younger cohorts [15]. Similarly, mild elevations in PAP are frequently observed in obese individuals [14]. These physiological variations highlight the limitations of absolute pressure thresholds and emphasize the need for an integrated, flow-adjusted interpretation of pulmonary haemodynamics—particularly during stress testing—to accurately distinguish normal physiology from early or latent pathological disease.

### 3.1. Recommended Indications for Stress Echocardiography

Stress echocardiography is recommended for diagnostic evaluation in the following clinical scenarios:

• Patients with unexplained exertional dyspnoea or exercise intolerance and normal or borderline resting echocardiographic findings;
• Patients with intermediate echocardiographic probability of pulmonary hypertension at rest;
• Patients with connective tissue diseases, particularly systemic sclerosis, with symptoms or borderline resting pulmonary pressures;
• Individuals with suspected exercise-induced pulmonary hypertension, including those with disproportionate symptoms relative to resting haemodynamics;
• Patients with suspected heart failure with preserved ejection fraction in whom differentiation between pre-capillary and post-capillary mechanisms during exercise is clinically relevant;
• First-degree relatives of patients with heritable or idiopathic pulmonary arterial hypertension, particularly when exertional symptoms are present.

### 3.2. Potential Indications for Stress Echocardiography

Stress echocardiography may be considered in selected cases where additional functional or haemodynamic information is expected to influence diagnostic decision-making:

• As part of risk stratification in patients with established pulmonary hypertension, particularly to assess right ventricular contractile reserve;
• In patients with borderline pulmonary pressures at rest in whom invasive haemodynamic testing is being contemplated;
• In patients unable to undergo immediate invasive testing, where non-invasive functional assessment may help guide referral timing;
• In selected athletes or highly conditioned individuals with symptoms and equivocal resting findings, when physiological adaptation must be distinguished from pathology.

### 3.3. Non-Recommended Settings for Stress Echocardiography

Stress echocardiography is generally not recommended in the following settings:

• Patients with overt pulmonary hypertension clearly established at rest, where stress testing is unlikely to alter diagnosis;
• Patients with poor acoustic windows in whom reliable Doppler assessment cannot be obtained despite optimization;
• As a standalone diagnostic test for pulmonary hypertension without planned integration into a structured diagnostic pathway;
• In patients with clinical instability, uncontrolled arrhythmias, or contraindications to exercise or pharmacological stress.

## 4. Recommended Stress Echocardiography Protocol

### 4.1. Stress Modality

Exercise stress echocardiography using a semi-supine bicycle ergometer is the preferred modality for evaluating pulmonary haemodynamics. This approach enables continuous Doppler imaging during incremental workloads, allowing real-time assessment of pulmonary pressures, cardiac output, and right ventricular functional responses [16,17]. Treadmill exercise is less suitable due to delays between exercise cessation and image acquisition, which may underestimate peak pulmonary pressures.

When physical exercise is contraindicated or not feasible, pharmacological stress with low-to-moderate-dose dobutamine (up to 20 μg/kg/min) may be used as an alternative to provoke haemodynamics stress while minimizing non-physiological effects such as excessive systemic vasodilation or pulmonary vasomotor changes [16,17,18]. Exercise stress remains the preferred modality because it preserves the physiological coupling between cardiac output, pulmonary vascular recruitment, and ventilatory responses. In contrast, dobutamine produces predominantly inotropic and chronotropic stimulation under resting loading conditions, which may result in haemodynamic patterns that are not fully comparable to dynamic exercise. Consequently, normal reference values and pressure–flow relationships during dobutamine stress are less well established, and diagnostic accuracy for detecting early pulmonary vascular disease may differ. Dobutamine stress should therefore be considered primarily when exercise testing is not feasible and interpreted with appropriate caution.

### 4.2. Imaging Protocol and Timing

A comprehensive protocol requires data acquisition at rest and peak stress. When feasible, intermediate workload stages are encouraged, as they provide valuable insights into pressure–flow relationships (e.g., mPAP/cardiac output [CO] slope), though not mandatory for basic diagnostic interpretation [15,16,17]. All images and Doppler signals must be obtained with optimized acoustic windows and precise beam alignment to ensure reliable measurements.

To enhance protocol standardization and reproducibility across laboratories, we propose a practical semi-supine bicycle workload framework for adults. The objective is to achieve a symptom-limited, incremental exercise test of approximately 8–12 min, which balances physiological stress with adequate time for Doppler acquisition. Workload progression should allow stable imaging at each stage while avoiding abrupt haemodynamic transitions that may compromise pressure–flow assessment. The following table provides a structured yet adaptable approach based on expected functional capacity.

Workload increments should be individualized based on age, baseline functional capacity, symptoms, and comorbidities. In low-capacity or frail patients, smaller steps (5–10 W) with longer stages (approximately 3 min) enhance Doppler feasibility and reduce premature termination (Table 1). In fitter subjects, larger increments are acceptable as long as sufficient time is allowed for TRV and LVOT VTI acquisition (Table 1).

The objective is to preserve incremental exercise physiology rather than achieve a predetermined peak workload. Intermediate stages are particularly useful when assessing pressure–flow relationships (e.g., mPAP/CO slope). At minimum, comprehensive data should be collected at rest and peak stress. Standard exercise test termination criteria for safety should always be applied.

Heart rate targets (e.g., ≥85% of predicted maximal) can be documented but should not serve as mandatory end points, particularly in patients receiving β-blocker therapy or those undergoing symptom-limited protocols. Haemodynamic interpretation should emphasize the workload achieved and the Doppler-derived pressure–flow responses rather than absolute heart rate thresholds.

### 4.3. Mandatory Haemodynamics and Functional Measurements

The following parameters should be evaluated at rest and peak stress:

• Peak TRV: Obtained via multi-window continuous-wave Doppler interrogation;
• Heart rate;
• Stroke volume: Calculated from left ventricular outflow tract diameter (measured at baseline) and velocity–time integral at each stage;
• Cardiac output: Calculated as heart rate × stroke volume;
• Right ventricular systolic function and contractile reserve: Assessed using tricuspid annular plane systolic excursion (TAPSE) and/or tissue Doppler S′ velocity;
• Left ventricular filling pressures: Including E/e′ ratio to aid differentiation of pre- vs. post-capillary components.

Systolic pulmonary artery pressure (sPAP) is estimated from peak TRV using the simplified Bernoulli equation (4 × TRV^2^) plus estimated right atrial pressure (RAP) (from baseline IVC parameters). Mean pulmonary artery pressure (mPAP) may be approximated using validated Doppler-derived formulae or full spectral envelope tracing when feasible [9,19].

All Doppler measurements should be averaged over at least three consecutive cardiac cycles at rest and peak stress to enhance accuracy and reproducibility.

### 4.4. Optional and Adjunct Measurements

• Contrast-enhanced Doppler imaging: Recommended to enhance tricuspid regurgitation signal quality in suboptimal windows, with caution regarding potential overestimation of pressures [9,19];
• Right ventricular outflow tract acceleration time: A useful complementary flow-based index when TRV is unreliable or unavailable [9,19].

## 5. Diagnostic Interpretation and Haemodynamic Phenotyping in Stress Echocardiography for Suspected Pulmonary Hypertension

### 5.1. Principles of Interpretation

Stress echocardiography in suspected PH must recognize the intrinsically flow-dependent nature of pulmonary haemodynamics. Absolute pulmonary artery pressure values during exercise alone are inadequate to define pathology and may lead to misclassification, especially in high-flow physiological states [6]. Interpretation should therefore prioritize the pulmonary pressure–cardiac output relationship, alongside evaluation of right ventricular functional reserve and left heart filling pressures.

The reintroduced definition of exercise pulmonary hypertension (mPAP/CO slope > 3 mmHg/L/min) is based exclusively on invasive haemodynamic measurements obtained during right heart catheterization [2,8]. Doppler-derived echocardiographic estimates of pulmonary pressure and cardiac output during exercise cannot replicate invasive measurements in terms of precision, temporal resolution, or reproducibility [2,8]. Consequently, non-invasive assessments of pressure–flow relationships should not be interpreted as diagnostic of exercise pulmonary hypertension but rather as physiological screening tools that may raise suspicion and support the need for invasive confirmation.

### 5.2. Primary Diagnostic Markers

The hallmark of an abnormal pulmonary vascular response is an excessive rise in pulmonary artery pressure that is disproportionate to the augmentation in cardiac output. This pattern may reflect potential overestimation of pressures [9,19] and is characterized by:

• A steep pulmonary pressure–flow relationship, indicated by an excessive increase in estimated mean pulmonary arterial pressure relative to cardiac output during stress;
• Failure of pulmonary vascular resistance to decrease appropriately with increasing flow.

When cardiac output measurements are available, pressure–flow indices provide superior diagnostic discrimination compared with absolute pressure thresholds. Therefore, fixed cut-off values of pulmonary artery pressure during exercise should not be used in isolation for diagnostic decision-making [20,21,22,23,24].

### 5.3. Supportive Echocardiographic Features

**Figure 2:**
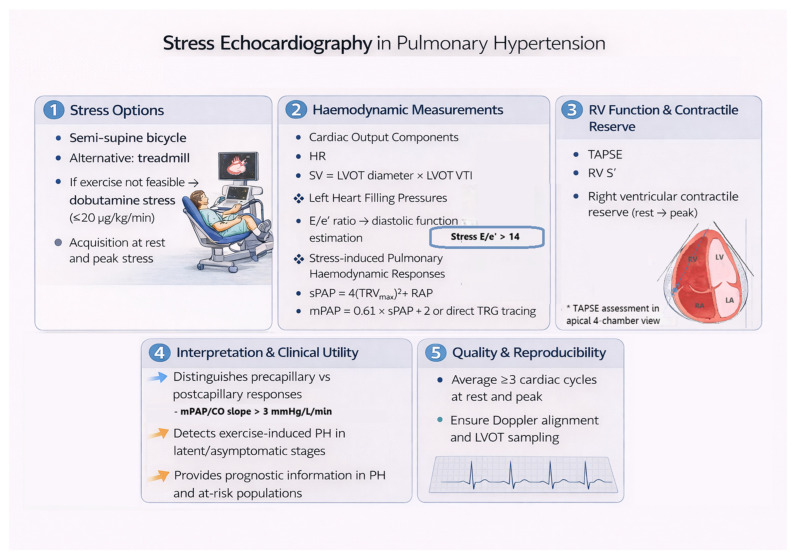
Stress echocardiography in PH. (**1**) Stress echocardiography options, with semi-supine bicycle exercise as the preferred method, treadmill exercise and dobutamine stress as alternatives. Data acquisition at rest and peak stress. (**2**) Key hemodynamic measurements during stress, including cardiac output components, estimation of left heart filling pressures (E/e′), and assessment of pulmonary pressures from tricuspid regurgitation-based indices. (**3**) Assessment of right ventricular systolic function and contractile reserve using stress-induced changes in TAPSE, RV S′ (between rest and peak stress). (**4**) Clinical interpretation of stress echocardiography, including differentiation between pre-capillary and post-capillary responses, detection of exercise-induced pulmonary hypertension, and prognostic stratification in at-risk populations. (**5**) Quality and reproducibility measures, averaging over 3 cardiac cycles and consistent Doppler alignment and sampling.

These ancillary findings strengthen suspicion of pathological haemodynamics when combined with abnormal pressure–flow behavior (Figure 2) [25,26,27,28]:

• Reduced right ventricular contractile reserve (e.g., attenuated increase in TAPSE and/or tissue Doppler S′ velocity during stress);
• Pulmonary artery pressure elevation disproportionate to exercise workload or intensity;
• Abnormal rise in left ventricular filling pressures (e.g., pronounced increase in E/e′ ratio).

Supportive features are not diagnostic in isolation but aid differentiation of pathological from physiological adaptations.

### 5.4. Quality Requirements, Feasibility, and Technical Considerations

Stress echocardiography for the assessment of pulmonary haemodynamics and right ventricular function is feasible in the majority of patients when performed using standardized protocols in experienced centers. Large feasibility studies have demonstrated high success rates for right ventricular functional assessment during stress, particularly for parameters reflecting right ventricular contractile reserve, such as tricuspid annular plane systolic excursion (TAPSE) and tissue Doppler S′ velocity, Table 2 [29].

In contrast, Doppler-based estimation of pulmonary pressures is technically more challenging at peak exercise. While tricuspid regurgitation velocity (TRV) is reliably obtained in most patients at rest, its feasibility frequently decreases during maximal stress due to hyperventilation, tachycardia, respiratory motion, and lung artefacts. Reported success rates vary across centers and protocols, Table 2 [29,30], meaning that pressure estimation based solely on TRV may not be achievable in all patients at peak workloads. In addition, inter- and intra-observer variability of TRV measurements—particularly during peak exercise—represents an important limitation that may affect reproducibility in routine clinical practice, further underscoring the need for standardized acquisition protocols and experienced operators [10,29].

Accurate haemodynamic assessment requires high-quality continuous-wave Doppler signal acquisition with careful optimization of imaging windows and beam alignment. Multi-window interrogation should be routinely employed to maximize the likelihood of obtaining a complete and well-defined TRV spectral envelope. Measurements derived from incomplete or poorly defined Doppler signals should not be used for diagnostic interpretation [9,18].

When tricuspid regurgitation signals are inadequate, contrast-enhanced Doppler imaging may improve feasibility and signal quality [31]. However, contrast use may result in overestimation of pulmonary pressures due to spectral signal broadening and should therefore be interpreted with caution and in conjunction with flow-based indices. In such cases, right ventricular outflow tract acceleration time represents a useful complementary parameter, offering high feasibility during exercise and providing additional flow-dependent information when tricuspid regurgitation velocity cannot be reliably obtained.

Estimation of RAP during stress represents a further technical limitation. Inferior vena cava-based estimates are often unreliable at peak exercise, and the assumption of constant RAP from rest to stress may lead to underestimation of true pulmonary artery pressure, particularly in patients with heart failure or post-capillary hemodynamic patterns.

In cases where TRV-based pressure estimation is unreliable, alternative flow-based indices provide valuable complementary information. Right ventricular outflow tract acceleration time (RVOT-AcT) offers excellent feasibility at rest and during stress, with strong correlation to invasively measured pulmonary artery pressure and high reproducibility [32]. Its flow-dependent characteristics make it a useful adjunctive parameter when conventional pressure measurements are limited.

The feasibility and diagnostic reliability of stress echocardiography are strongly influenced by operator expertise and laboratory experience. Accordingly, stress echocardiography for pulmonary hemodynamics assessment should be performed in centers with specific expertise in right heart imaging and pulmonary vascular disease, using standardized acquisition, interpretation, and reporting protocols, as recommended by current echocardiographic guidelines (Figure 2) [9,18].

Several methodological constraints further limit the diagnostic equivalence of stress echocardiography to invasive haemodynamic testing. Continuous-wave Doppler acquisition of tricuspid regurgitation velocity during exercise is susceptible to signal dropout, suboptimal beam alignment, tachycardia-related envelope shortening, and respiratory motion [2,16,19,31]. Cardiac output estimation relies on left ventricular outflow tract (LVOT) diameter measured at rest and velocity–time integral (VTI) recordings obtained during stress, introducing cumulative measurement variability [2,16,19,31].

Stroke volume is calculated from LVOT cross-sectional area—derived from a single baseline LVOT diameter measurement assuming circular geometry and constant diameter during exercise—and the LVOT VTI obtained at peak workload. Because LVOT diameter is squared in the area calculation, even small measurement inaccuracies may be amplified [9,10]. At high heart rates, beat-to-beat variability related to respiration, preload fluctuations, and tachycardia may further reduce VTI reproducibility. Doppler beam alignment with LVOT flow becomes more challenging during peak exercise due to patient motion and hyperventilation, potentially leading to velocity underestimation [9,10]. Since cardiac output is calculated as stroke volume × heart rate, variability in stroke volume measurement may be magnified at higher workloads.

In addition, right atrial pressure during exercise is typically assumed to remain constant, which may not reflect true haemodynamic behavior. When intermediate stages are used to calculate pressure–flow slopes, small measurement deviations may disproportionately influence slope estimation [9,10]. These sources of variability underscore that non-invasive slope assessment should be interpreted cautiously and within experienced laboratories, and should not be considered interchangeable with invasive measurement.

## 6. Clinical Scenarios with High Diagnostic Yield

Stress echocardiography demonstrates the highest diagnostic yield in clinical settings characterized by exertional symptoms, borderline or inconclusive resting echocardiographic findings, and an increased pre-test probability of pulmonary vascular or cardiac involvement. In such scenarios, dynamic hemodynamics assessment may reveal abnormal pulmonary pressure–flow responses and right ventricular functional impairment that are not evident at rest [33,34].

### 6.1. Systemic Sclerosis and Connective Tissue Diseases

Patients with systemic sclerosis and other connective tissue diseases are at increased risk for the development of pulmonary arterial hypertension. In these populations, resting echocardiography may remain normal or borderline despite early pulmonary vascular involvement. Stress echocardiography may unmask abnormal pulmonary haemodynamics responses during exercise, including disproportionate increases in pulmonary artery pressure and impaired right ventricular reserve, thereby supporting earlier identification of pulmonary vascular disease and guiding referral for invasive haemodynamics confirmation [35,36,37,38].

### 6.2. Relatives of Patients with Heritable or Idiopathic Pulmonary Arterial Hypertension (PAH)

First-degree relatives of patients with heritable or idiopathic pulmonary arterial hypertension represent a recognized at-risk population. In this setting, stress echocardiography may identify exaggerated pulmonary pressure responses or reduced pulmonary vascular reserve during exercise, even in the absence of resting abnormalities. These findings may provide additional functional information in symptomatic individuals or those with borderline resting echocardiographic parameters [39].

### 6.3. Unexplained Exertional Dyspnea with Normal or Borderline Resting Echocardiography

In patients presenting with exertional dyspnea or exercise intolerance that is disproportionate to resting echocardiographic findings, stress echocardiography may help uncover latent pulmonary vascular or right ventricular dysfunction. This scenario represents one of the most frequent and clinically relevant indications for stress echocardiography, particularly when resting pulmonary pressures are normal or only mildly elevated, Table 2 [31,33].

### 6.4. Suspected Heart Failure with Preserved Ejection Fraction

Stress echocardiography provides valuable insight in suspected HFpEF, particularly when resting haemodynamics are normal or borderline. The hallmark abnormality is impaired LV diastolic reserve: during exercise, reduced compliance and delayed relaxation lead to a disproportionate rise in LV filling pressure (PAWP), even when resting values are normal [14,28].

Simultaneous invasive–echocardiographic studies have shown that exercise-induced PAWP elevation represents the dominant early haemodynamic abnormality in HFpEF. In patients with unexplained exertional dyspnoea and preserved ejection fraction, exercise right heart catheterisation reveals marked increases in PAWP despite normal resting pressures, Table 2 [28]. Exercise E/e′ correlates with invasively measured filling pressures, supporting stress echocardiography as a screening and phenotyping tool rather than a diagnostic substitute for catheterisation, Table 2 [28,40,41].

The rise in PAWP drives a secondary increase in mPAP, indicating a predominantly post-capillary pattern that may evolve into combined post- and pre-capillary disease due to pulmonary vascular remodeling [14]. RV–pulmonary arterial (RV–PA) uncoupling during exercise—reflecting limited RV contractile reserve relative to increased afterload—further contributes to exertional intolerance and adverse haemodynamic phenotype [42].

Stress echocardiography helps assess these mechanisms through integrated interpretation:

• A marked increase in E/e′ accompanied by rising pulmonary pressure suggests a post-capillary HFpEF response.
• An abnormal pressure–flow slope with minimal E/e′ change raises suspicion of pulmonary vascular involvement.
• A blunted TAPSE or S′ response implies impaired RV–PA coupling.

While stress echocardiography cannot directly measure PAWP or RV–PA coupling ratios, it enhances non-invasive haemodynamic assessment and identifies patients who may benefit from confirmatory exercise right heart catheterization.

### 6.5. Athletes and Highly Conditioned Individuals

In selected athletes or highly trained individuals with exertional symptoms and equivocal resting echocardiographic findings, stress echocardiography may assist in differentiating physiological hemodynamic adaptation from pathological responses. Interpretation in this population requires careful integration of workload, cardiac output, and pressure–flow relationships and should be performed in experienced centers [43,44].

### 6.6. Pulmonary Hypertension and Valvular Heart Disease

Pulmonary hypertension commonly accompanies left-sided valvular heart disease, particularly mitral and aortic valve pathology, and carries significant diagnostic and prognostic implications. Stress echocardiography may provide incremental information by unmasking exercise-induced pulmonary pressure elevation, clarifying the post-capillary contribution to pulmonary hypertension, and identifying patients who exhibit disproportionate haemodynamic responses despite only moderate resting valve disease [45,46,47]. In this setting, the combined assessment of tricuspid-regurgitation-derived pressure changes, left-ventricular filling pressures (E/e′), and RV contractile reserve during stress can support clinical decision-making and optimize the timing of intervention [45,46,47]. Interpretation, however, requires careful integration with valve severity, loading conditions, and exercise capacity, since pulmonary pressure responses in this population are strongly flow-dependent.

## 7. Stress Echocardiography in Pediatric Pulmonary Hypertension

Pulmonary hypertension in children and adolescents presents unique challenges due to underlying etiologies (e.g., congenital heart disease, developmental pulmonary vascular disease, idiopathic/heritable PAH), differing physiology (e.g., higher pulmonary vascular reserve in early life), and age-specific considerations in assessment and management. While the 2022 ESC/ERS Guidelines emphasize a comprehensive diagnostic approach including right heart catheterization as the gold standard, non-invasive tools like stress echocardiography are gaining interest for functional evaluation in selected pediatric cases [48,49].

In children, resting echocardiography often underestimates disease severity in early or latent PH, similar to adults, due to preserved vascular compliance and adaptive RV responses. Exercise or pharmacological stress echocardiography can provide dynamic insights into pulmonary pressure responses, RV contractile reserve, and biventricular function during increased cardiac output—potentially unmasking subclinical abnormalities or guiding risk stratification [50,51,52].

### 7.1. Recommended or Potential Indications in Pediatrics

• Children with congenital heart disease (e.g., repaired septal defects, univentricular physiology, or pulmonary artery anomalies) and exertional symptoms or borderline resting pulmonary pressures;
• Symptomatic patients with suspected idiopathic/heritable PAH or connective tissue disease-associated PH, where resting findings are inconclusive;
• Assessment of RV–pulmonary arterial coupling and functional reserve in established pediatric PH for risk stratification or therapy monitoring;
• Differentiation of physiological adaptations from pathology in active children or adolescents.

### 7.2. Protocol Considerations in Children

• Prefer semi-supine bicycle ergometer (adapted workloads starting low, e.g., 0.5–1 W/kg increments) for continuous imaging; treadmill may be used in cooperative older children;
• Pharmacological stress (low-dose dobutamine) as alternative if exercise is not feasible;
• Mandatory parameters mirror adult protocols (TRV, CO estimation, TAPSE/S′, E/e′), with emphasis on RVOT acceleration time if TRV is challenging;
• Feasibility is high in experienced pediatric echocardiography labs, but cooperation, sedation needs (rarely), and motion artifacts require specialized expertise [47].

Interpretation follows adult principles: prioritize pressure–flow relationships over absolute pressures, integrate with clinical context, and avoid isolated fixed cut-offs. Abnormal responses (e.g., steep mPAP/CO slope, blunted RV reserve) should prompt referral for invasive confirmation.

## 8. Conclusions

Stress echocardiography serves as a valuable non-invasive adjunct in the diagnostic evaluation of pulmonary hypertension when applied selectively within a structured, physiology-based framework. While right heart catheterisation remains the gold standard for definitive diagnosis and haemodynamic characterization, resting transthoracic echocardiography alone is often insufficient in patients with early/latent disease, borderline findings, or symptoms disproportionate to resting haemodynamics.

By enabling dynamic assessment of pulmonary pressure responses, cardiac output augmentation, and right ventricular functional reserve during exercise, stress echocardiography provides incremental diagnostic and prognostic information beyond resting evaluation. Its primary utility lies in identifying abnormal pressure–flow relationships (e.g., excessive mPAP rise relative to cardiac output), impaired pulmonary vascular reserve, and reduced right ventricular contractile reserve—rather than relying on absolute pulmonary artery pressure thresholds during exercise.

Contemporary interpretation must adopt an integrated, flow-adjusted approach that incorporates workload, cardiac output, ventricular interaction, and clinical context. Fixed exercise pulmonary pressure cut-offs should not be used in isolation, given the substantial influence of physiological, demographic, and constitutional factors on pulmonary haemodynamics.

This aligns with the 2022 ESC/ERS Guidelines’ reintroduction of exercise pulmonary hypertension, defined invasively by an mPAP/CO slope > 3 mmHg/L/min. Stress echocardiography cannot establish this diagnosis non-invasively but may identify abnormal pressure–flow patterns that warrant referral for confirmatory invasive haemodynamic assessment [2].

When performed in experienced centers using standardised protocols and meticulous Doppler optimisation, stress echocardiography can help differentiate pre-capillary from post-capillary (or mixed) mechanisms, refine haemodynamic phenotyping, and guide appropriate referral for invasive confirmation—including exercise right heart catheterisation when clinically indicated. It is not a standalone diagnostic modality but a complementary tool that enhances patient selection for invasive testing and supports earlier identification of clinically relevant disease in high-yield scenarios (e.g., systemic sclerosis, heritable PAH relatives, unexplained exertional dyspnoea, and suspected HFpEF).

In summary, applied selectively in selected patients, according to recommended protocols, and interpreted through pressure–flow-based principles, stress echocardiography occupies a well-defined and meaningful role in the modern diagnostic pathway for pulmonary hypertension.

## Figures and Tables

**Table 1:** Standardized semi-supine bicycle workload progression for adult stress echocardiography in pulmonary haemodynamic assessment.

Profile	Start Load	Increment	Stage Duration	Target Exercise Time	Practical Notes
**Low capacity/Symptomatic** (frail *,* advanced age, HFpEF suspicion, severe dyspnoea)	10–20 W	5–10 W	2–3 min	8–12 min	Use smaller steps to improve pressure–flow assessment and allow Doppler acquisition. Consider 3-min stages if TRV or LVOT VTI acquisition is challenging.
**Moderate capacity** (typical patient)	20–25 W	10–15 W	2–3 min	8–12 min	Common “default” protocol. Provides a balanced progression suitable for most patients.
**High capacity/Fit** (younger, athletic, mild symptoms)	25–50 W	15–25 W	2–3 min	8–12 min	Larger increments acceptable. Ensure Doppler acquisition at each stage remains feasible. Useful for trained or minimally symptomatic individuals.

**Table 2:** Summary of Clinical Studies Evaluating Exercise-Induced Pulmonary Hypertension.

Author/Year	Subjects (n)	Type of Exercise	Protocol	Main Findings/Clinical Implication
Grünig et al., 2009 [38]	482 subjects (191 controls, 291 relatives of patients with PAH)	Supine bicycle and 120 min of normobaric hypoxia (FIO2 12%)	Exercise: Cycling at increasing workloads of 25 W every 2 min until max effort. Hypoxia: 120 min exposure, measuring TRV at 45, 90, and 120 min.	Showed that asymptomatic relatives of PAH patients frequently exhibit hypertensive responses to stress, suggesting a latent “pre-clinical” phenotype linked to genetic susceptibility.
Gargani et al., 2013 [35]	164 patients with SSc	Graded semi-supine bicycle ergometer	Workload increased by 25 Watts every 2 min	Demonstrated that exercise-induced PH (sPAP > 50 mmHg) in SSc aids in early diagnosis and correlates with diastolic dysfunction, even when resting pressures are normal.
Kusunose et al., 2015 [33]	78 patients (54 SSc, 16 SLE, 8 MCTD)	6 Minute Walking Test	Exercise protocol measuring ΔPASP	Proved that pulmonary pressure augmentation during a simple 6-min walk test predicts the future development of overt resting PH.
Claessen et al., 2016 [31]	61 subjects (19 athletes, 9 healthy non-athletes, 8 healthy BMPR2 mutation carriers, 5 patients with new or worsening dyspnea, 20 patients with chronic CTPH)	Semi-recumbent cycle ergometer (tilted 20–30°)	Workload started at 20 W and increased by 20 W every 2 min until exhaustion or symptom limitation	Validated echocardiographic mPAP/CO slope against invasive measurement. Established the cut-off of >3 mmHg/L/min for defining abnormal pulmonary vascular reserve.
Borlaug et al., 2016 [27]	74 subjects (50 HFpEF, 24 controls)	Supine bicycle (Simultaneous Invasive with RHC)	Workload started at 20 W for 5 min, then 10 W increments (3 min stages) to exhaustion	Demonstrated that impaired RV contractile reserve and abnormal RV-PA coupling during exercise are key determinants of symptoms in HFpEF, identifying it as a biventricular disorder.
Obokata et al., 2017 [28]	74 subjects (50 HFpEF, 24 NCD)	Supine bicycle (Simultaneous Invasive with RHC)	Workload started at 20 W for 5 min, then 10 W increments to exhaustion	Validated the Diastolic Stress Test. Showed that adding Exercise E/e’ (>14) to resting parameters significantly improves sensitivity and negative predictive value for diagnosing HFpEF.
Chen et al., 2019 [40]	34 HFpEF subjects	Supine electromagnetic braked cycle ergometer (Simultaneous Invasive with RHC)	Constant workload of 20 W for 6 min	Demonstrated that Stress E/e’ (but not resting E/e’) correlates significantly with invasive PCWP during exercise, validating its utility for identifying exercise-induced post-capillary PH
Wierzbowska- Drabik et al., 2019 [30]	102 subjects (33 with PH, 30 with CV risk factors, 39 healthy controls)	Cycle ergometer	Workload increased by 25 Watts every 2 min	Demonstrated that RVOT AcT is a feasible and reliable flow-dependent surrogate for assessing pulmonary hemodynamics during exercise, particularly when TR signal is suboptimal.
Ferrara et al., 2021 [29]	954 subjects (254 healthy controls, 40 elite athletes, 658 patients at risk, or with overt PH)	Semi-recumbent bike	Incremental workload of 25 Watts every 2 min	Confirmed the high feasibility of SE in a large multicenter cohort. Provided reference values for RV contractile reserve (TAPSE, S’) at peak stress.

Abbreviations: AcT, Acceleration Time; BMPR2, Bone Morphogenetic Protein Receptor type 2; CO, Cardiac Output; CTPH, Chronic Thromboembolic Pulmonary Hypertension; CV, Cardiovascular; FiO2, Fraction of Inspired Oxygen; HFpEF, Heart Failure with preserved Ejection Fraction; MCTD, Mixed Connective Tissue Disease; mPAP, Mean Pulmonary Arterial Pressure; NCD, Non-Cardiac Dyspnea; PAH, Pulmonary Arterial Hypertension; PASP/sPAP, Pulmonary Artery Systolic Pressure; PCWP, Pulmonary Capillary Wedge Pressure; PH, Pulmonary Hypertension; RHC, Right Heart Catheterization; RV, Right Ventricle; RV-PA, Right Ventricular-Pulmonary Arterial; RVOT, Right Ventricular Outflow Tract; S’, Systolic Tissue Doppler Velocity; SE, Stress Echocardiography; SLE, Systemic Lupus Erythematosus; SSc, Systemic Sclerosis; TAPSE, Tricuspid Annular Plane Systolic Excursion; TR (V), Tricuspid Regurgitation (Velocity); W, Watts.

## Data Availability

No new data were created or analyzed in this study. Data sharing is not applicable to this article.

## Abbreviations

The following abbreviations are used in this manuscript:

AcT	Acceleration time
BMI	Body mass index
CO	Cardiac output
ERS	European Respiratory Society
ESC	European Society of Cardiology
HFpEF	Heart failure with preserved ejection fraction
IVC	Inferior vena cava
LVOT	Left ventricular outflow tract
mPAP	Mean pulmonary arterial pressure
RA	Right atrial
RAP	Right atrial pressure
RHC	Right heart catheterization
RV	Right ventricular
RVOT	Right ventricular outflow tract
sPAP	Systolic pulmonary artery pressure
TAPSE	Tricuspid annular plane systolic excursion
TRV	Tricuspid regurgitation velocity
TTE	Transthoracic echocardiography
PAH	Pulmonary arterial hypertension
PAP	Pulmonary arterial pressure
PH	Pulmonary hypertension
PVR	Pulmonary vascular resistance
VTI	Velocity time integral
